# Enzymatic Hydrolysis and Fermentation of Pea Protein Isolate and Its Effects on Antigenic Proteins, Functional Properties, and Sensory Profile

**DOI:** 10.3390/foods11010118

**Published:** 2022-01-04

**Authors:** Verónica García Arteaga, Victoria Demand, Karolin Kern, Andrea Strube, Michael Szardenings, Isabel Muranyi, Peter Eisner, Ute Schweiggert-Weisz

**Affiliations:** 1 Fraunhofer Institute for Process Engineering and Packaging IVV, 85354 Freising, Germany; veronica.gart@gmail.com (V.G.A.); victoria.demand@gmx.de (V.D.); andrea.strube@ivv.fraunhofer.de (A.S.); isabel.muranyi@ivv.fraunhofer.de (I.M.); peter.eisner@ivv.fraunhofer.de (P.E.); 2 Center of Life and Food Sciences Weihenstephan, Technical University of Munich, 85354 Freising, Germany; 3 Fraunhofer Institute for Cell Therapy and Immunology IZI, 04103 Leipzig, Germany; karolin.kern@izi.fraunhofer.de (K.K.); michael.szardenings@izi.fraunhofer.de (M.S.); 4 ZIEL—Institute for Food & Health, Technical University of Munich, 85354 Freising, Germany; 5 Steinbeis-Hochschule, School of Technology and Engineering, 12489 Berlin, Germany; 6 Institute for Nutritional and Food Sciences, University of Bonn, 53115 Bonn, Germany

**Keywords:** pea protein isolate, lactic fermentation, *Lactobacillus plantarum*, enzymatic hydrolysis, functional properties, protein solubility, pea allergens, sensory properties, bitterness

## Abstract

Combinations of enzymatic hydrolysis using different proteolytic enzymes (papain, Esperase^®^, trypsin) and lactic fermentation with *Lactobacillus plantarum* were used to alter potential pea allergens, the functional properties and sensory profile of pea protein isolate (PPI). The order in which the treatments were performed had a major impact on the changes in the properties of the pea protein isolate; the highest changes were seen with the combination of fermentation followed by enzymatic hydrolysis. SDS-PAGE, gel filtration, and ELISA results showed changes in the protein molecular weight and a reduced immunogenicity of treated samples. Treated samples showed significantly increased protein solubility at pH 4.5 (31.19–66.55%) and at pH 7.0 (47.37–74.95%), compared to the untreated PPI (6.98% and 40.26%, respectively). The foaming capacity was significantly increased (1190–2575%) compared to the untreated PPI (840%). The treated PPI showed reduced pea characteristic off-flavors, where only the treatment with Esperase^®^ significantly increased the bitterness. The results from this study suggest that the combination of enzymatic hydrolysis and lactic fermentation is a promising method to be used in the food industry to produce pea protein ingredients with higher functionality and a highly neutral taste. A reduced detection signal of polyclonal rabbit anti-pea-antibodies against the processed protein preparations in ELISA furthermore might indicate a decreased immunological reaction after consumption.

## 1. Introduction

Peas (*Pisum sativum* L.) are increasingly used due to their sustainable production [1], economic benefits [2], high protein content (15–30%), and alleged low allergenicity. They belong to the legume family (Fabaceae) and their proteins are classified as salt-, water-, and ethanol soluble, corresponding to globulins, albumins, and prolamins, respectively [3].

Peas are not on the list of main allergens and do not need to be declared as allergenic in food products; however, two allergenic protein fractions from the storage proteins have been identified [4] and are recognized by the International Committee of Allergen Nomenclature as main pea globulin allergens. The allergen Pis s1 correspond to the mature vicilin (47–50 kDa) as well as to one of vicilin’s proteolytic fractions (32 kDa). The Pis s2 correspond to convicilin (67–70 kDa). The ability of allergen proteins (antigen) to cause an immune response (immunogenicity) depends on different factors, such as the antigen dose, exposure, and host genetic background [5], and thus, their ability to cause allergic reactions [6]. Moreover, pea allergens have shown homology between epitopes (recognition sites) from other legume allergens [7] and serological cross-reactivity has been proved [4,8,9]. Different methods to modify food allergens and their impact on food allergenicity have been reviewed [10].

Enzymatic hydrolysis is one of the most common methods used for this purpose and has been proven effective in allergen degradation of different legumes [11,12,13]. Modification of pea allergens by enzymatic treatment has been studied to a lesser extent. Pea protein isolate (PPI) treated with trypsin [14], Alcalase [15], flavourzyme, papain, and pepsin [16] have shown a reduced immunogenicity by means of ELISA methods. Frączek, Kostyra [14] found that a higher degree of hydrolysis resulted in a higher reduction in immunogenic potential. Moreover, changes in the molecular weight distribution of proteins are also known to affect functional and sensory properties. Partial hydrolysis was shown to increase protein solubility and emulsifying capacity; however, further hydrolysis reduced both [17,18,19]. Depending on the composition, the low molecular weight peptides formed during enzymatic hydrolysis can promote a bitter taste. The mechanism is not yet fully understood but mainly hydrophobic amino acid residues appear responsible [20].

For debittering of protein hydrolysates, fermentation has been widely studied [21,22,23,24,25]. Lactic acid bacteria reduced the bitterness of hydrolysates by releasing aminopeptidases cleaving hydrophobic amino acid residues [23]. There are several studies focusing on changes in the aroma profile of fermented pea, pea proteins, and pea products [26,27,28]; however, to our knowledge, there are no studies focusing on the debittering of pea protein hydrolysates by lactic fermentation.

The effects of fermentation on the functional properties have been studied for different legumes [21,29,30], and, to a lesser extent, for peas [31,32,33]. Moreover and to our knowledge, only one study has investigated the effects of fermentation on the antigenicity of pea flour [34].

The combination of enzymatic hydrolysis and microbial fermentation seems very promising for the production of low-allergenic and tasty functional food ingredients. A combination has been investigated for soy [22] and lupin protein isolate [35,36], but not yet for pea. For this reason, this study aimed to investigate the effects of combining enzymatic hydrolysis and fermentation on allergenic proteins (Pis s1 and Pis s2), as measured by SDS-PAGE and the ability of polyclonal sera to recognize antigens, functional properties and on the debittering and characteristic off-flavors of pea proteins. According to previous findings, papain, Esperase^®^, trypsin and *Lactobacillus plantarum* were selected for enzymatic hydrolysis [18] and fermentation [33], respectively. The specificity of an enzyme is determined by the arrangement of amino acids within the active site and the structure of the substrates. The acidification during fermentation could cause protein aggregation hiding protein parts from binding with the enzyme active site. Therefore, two sequences of the reactions, enzymatic treatment and fermentation, were investigated as the order of the method combination might be relevant for changes in the molecular weight distribution of the hydrolysates, functional properties and taste. Moreover, the treatments and the order of the method combination might also change the epitope binding sites and thus, the immunogenicity of pea allergenic proteins.

## 2. Materials and Methods

### 2.1. Materials

Pea seeds (*Pisum sativum* L., cultivar Navarro) were provided by Norddeutsche Pflanzenzucht Hans-Georg-Lembke KG (Holtsee, Germany). Trypsin and Esperase^®^ 8.0 L were obtained from Sigma-Aldrich (Munich, Germany). Papain was from Carl Roth GmbH (Karlsruhe, Germany). *L. plantarum* (DSM 20174) was purchased from the German collection of microorganisms and cell cultures (Deutsche Sammlung von Mikroorganismen und Zellkulturen, Germany). Broad Range™ Unstained Protein Standard, 4–20% Criterion™ TGX stain-free™ precast polyacrylamide gels, Coomassie Brilliant Blue R-250 were from Bio-Rad Laboratories GmbH (Feldkirchen, Germany). Sodium dihydrogen phosphate, sodium dodecyl sulfate, sodium tetraborate decahydrate, o-phthaldialdehyde, and sodium monohydrogen phosphate were purchased from Sigma-Aldrich (Munich, Germany). All chemicals used in this study were of analytical grade unless otherwise indicated.

### 2.2. Production of Pea Protein Isolate

Pea flour was prepared by dehulling, splitting and impact-milling pea seeds as described by García Arteaga, Leffler [33]. The pea protein isolation was performed according to García Arteaga, Apéstegui Guardia [18]. Briefly, an alkaline protein extract (pH 8.0) was adjusted to pH 4.5 for protein isoelectric precipitation. The precipitated proteins were neutralized, pasteurized (70 ± 2 °C) for 2 min and spray-dried.

### 2.3. Pea Protein Isolate Modification

The PPI was treated by enzymatic treatment, microbial fermentation or a combination of both. Table 1 shows the specific conditions for the enzyme preparations and microbial strain. The combination experiments were carried out as follows: enzymatic hydrolysis with the individual enzyme preparations followed by fermentation (HyF), and fermentation followed by hydrolysis (FdH), and are presented in Table 2. A 9% (*w*/*w*) PPI dispersion in DI water was homogenized using an Ultra-Turrax (IKA^®^ Werke GmbH & Co KG, Staufen, Germany) for 90 s at 11,000 rpm and pasteurized at 80 °C for 10 min. The pH and temperature were adjusted to the optimal conditions (Table 1) prior to the addition of enzymes or of *L. plantarum* in each treatment. The pH was adjusted using 3.0 mol/L hydrochloric acid or 3.0 mol/L sodium hydroxide. Inactivation of enzymes or microorganisms was performed at 90 °C for 10 min before proceeding to the next treatment or finalizing the experiment. The denatured enzyme and the inactivated microbial cells were not removed from the samples. The final samples were neutralized (pH 7.0) at room temperature, lyophilized and grinded for 10 s at 7500 rpm (Grindomix GM200, Retsch GmbH, Haan, Germany). An untreated PPI dispersion was used as reference. Samples of each treatment were prepared in duplicate.

#### 2.3.1. Fermentation

##### Growth and Culture Conditions

To optimally cultivate *L. plantarum* strains, a late exponential growth phase was chosen. Briefly, a 200-μL aliquot of *L. plantarum* in MRS (De Man, Rogosa, and Sharpe) covered with 50 μL sterile paraffin oil was incubated using a microplate reader (Synergy HTX, BioTek Instruments GmbH, Waldbronn, Germany). The OD was measured every 15 min at a wavelength of 600 nm. The exponential phase lasted approximately from the 11 h until the 24 h since beginning of fermentation; thus, a late exponential phase was selected at 18 h to obtain inocula of *L. plantarum*.

##### Determination of Viable Cell Counts for Inoculum and after Fermentation

The *L. plantarum* was incubated in MRS-broth for 18 h at 30 °C under anaerobic conditions. Serial dilutions were used for the determination of viable bacteria cell and OD measurements to select the OD corresponding to a viable cell count of a 7-log colony forming units per milliliter per sample (CFU/mL). The OD 0.1 was used as reference for liquid cultured aliquots before each fermentation. The log CFU/mL of fermented samples were determined at the beginning and the end of the fermentation on MRS agar from 100 μL of diluted sample.

##### Fermentation of PPI Dispersions

The pasteurized PPI dispersions or inactivated PPI hydrolysates were transferred into sterile 2-L Schott flasks. Prior to inoculation, the solutions were adjusted to pH 6.5 and cooled down to 30 °C before 0.5% (*w*/*v*) glucose was added. The aliquot taken for CFU determination represented the initial viable cell number t = 0 h after 10 min inoculation. The flasks were flushed with nitrogen to achieve anaerobic conditions and the fermentation was carried out for 24 h without stirring. The pH was assessed after 24 h. After inactivation, the HyF samples were cooled to room temperature, neutralized, and lyophilized. For the FdH samples, the inactivated fermented solutions were adjusted to the optimal conditions of each enzyme.

#### 2.3.2. Enzymatic Hydrolysis

The pasteurized PPI dispersions or inactivated fermented PPI were transferred to thermostatically controlled stainless-steel reactors and the optimal conditions for each enzyme were set. The enzyme to substrate ratio was calculated based on the protein content. The hydrolysis was carried out for 2 h with constant stirring (80 rpm) using an agitator (R50-20D, Phoenix Instruments GmbH, Garbsen, Germany) and maintaining optimal conditions. After inactivation, the HyF samples were cooled and adjusted to the optimal conditions for fermentation. The FdH samples were cooled to room temperature, neutralized, and lyophilized. The sample codes are shown in Table 2.

### 2.4. Chemical Composition

The dry matter content (105 °C), ash content (950 °C) and protein content (N × 6.25) were analyzed according to AOAC Official Methods [37,38] by means of a thermogravimetric method (TGA 701, Leco Instruments, Germany) and the Dumas combustion method (TruMac N, Leco Instruments, Mönchengladbach, Germany), respectively.

### 2.5. Determination of Protein Degradation

#### 2.5.1. Molecular Weight Distribution

The molecular weight distribution was analyzed by sodium dodecyl sulfate polyacrylamide gel electrophoresis (SDS-PAGE) according to Laemmli [39] with slight modifications and as described in detail in García Arteaga, Apéstegui Guardia [18]. Briefly, 5 μg/μL protein solution (based on dry matter) was prepared in 1× reducing buffer (50% (*v*/*v*) 2× Tris-HCl reducing buffer, 50% (*v*/*v*) phosphate buffer (pH 7)). The samples were heated (95 °C, 5 min) prior to centrifugation at 12,045× *g* for 3 min (MiniSpin, Eppendorf AG, Hamburg, Germany). An aliquot of 3 μL of the supernatants was added into the gel pocket of the Bio-Rad 4–20% Criterion™ TGX Stain-Free™ Precast Gels. The Broad Range™ Unstained Protein Standard was used as the molecular weight marker. The running time was 30 min, followed by staining using Coomassie Brilliant Blue R-250. Finally, gel images were obtained using an EZ Imager (Gel Doc™ EZ Imager, Bio-Rad Laboratories, Feldkirchen, Germany). SDS-PAGE was performed in duplicate, with each sample being prepared two times independently.

#### 2.5.2. Degree of Hydrolysis

The degree of hydrolysis (DH) was determined according to Nielsen, Petersen [40] using o-phthaldialdehyde (OPA). The DH was calculated based on the total number of peptide bonds per protein equivalent (h_tot_). The constant values used for α (degree of dissociation of the α-amino group), β (slope of calibration through linear regression) and htot factor were 1.0, 4.0, and 8.0, respectively, according to theoretical general values for unexamined raw material [40]. The DH was calculated according to the following equations:

$$Serine\text{-}NH_{2}=\frac{Abs_{sample}-Abs_{blank}}{Abs_{standard}-Abs_{blank}}\times 0.951\frac{meqv}{L}\times \frac{V_{sample}\times 100}{m_{sample}\times PC_{sample}}$$

*Serine*-*NH*_2_ = meqv serine-NH_2_/g protein;
*Abs_sample_* = sample absorbance value;
*Abs_blank_* = blank absorbance value;
*Abs_standard_* = standard absorbance value;
*V_sample_* = volume of sample solution (L);
*m_sample_* = weight of sample (g);
*PC_sample_* = protein content of sample (%);

$$h=\frac{Serine\text{-}NH_{2}-\beta }{\alpha }$$

*Serine*-*NH*_2_ = meqv serine-NH_2_/g protein;
*h* = number of hydrolyzed peptide bonds;
*β* = slope of calibration through linear regression;
*α* = degree of dissociation of the α-amino group;

$$DH=\frac{h}{h_{tot}}\times 100$$

*DH* = degree of hydrolysis (%)
*h* = number of hydrolyzed peptide bonds;
*h_tot_* = total number of peptide bonds per protein equivalent.

The sample preparation was performed in duplicate and each prepared sample was measured in triplicate.

#### 2.5.3. Gel Filtration Chromatography

Two grams of untreated and treated samples were solubilized in 2 mL of 50 mM Tris-HCl and 100 mM KCl, pH 7.5. Samples were centrifuged in an Eppendorf centrifuge 5424 R at 20,000× *g*. Supernatant (1.6 mL) was applied to Superdex 200 gel filtration column (26/600, GE Healthcare; 60 cm × 26 mm) using ÄKTA avant System. The sample was processed at a flow rate of 2 mL/min in 50 mM Tris-HCl and 100 mM KCl, pH 7.5. Peak eluate fractioning was used to collect the eluate in 2.5-mL fractions. Elution was monitored at 280 nm. On average 74 fractions were collected.

#### 2.5.4. Generation of Polyclonal Rabbit Sera

The immunization of three rabbits (“Continental Giant”) with a suspension of the untreated PPI powder was performed by a certified external supplier (Seramun Diagnostica GmbH, Heidesee, Germany). Three rabbits are required to obtain a complete coverage of all proteins. A basic immunization with 1 mg and Complete Freund’s Adjuvant was followed by one booster injection on day 21 using 0.5 mg in combination with Incomplete Freund’s Adjuvant. The serum was recovered 7 days after the booster injection. Final sera showed at >1,000,000 dilution >5× binding to PPI compared to the pre-immune serum.

#### 2.5.5. Immunogenicity Measured by Enzyme-Linked Immunosorbent Assay (ELISA)

Purified sample fractions were measured in duplicate by indirect ELISA. MaxiSorp 96-well immuno plates (Life Technologies) were coated by adding 100 µL of gel filtration fractions to each well. The plates were incubated at 4 °C for 20 h. The wells were emptied and 100 µL 5% NFDM (blocking buffer) in PBS was added to each well. The plates were incubated for 1 h at 4 °C. After 3× washing with 0.1% Tween/PBS, the plates were incubated with the rabbit sera immunized with PPI (1:2000 in blocking buffer) at 4 °C for 1 h. Another washing step with 0.1% Tween/PBS was performed. Moreover, 100 µL/well the detection antibody (Goat-anti-rabbit IgG, Dianova 111-035-003, 1:5000 in blocking buffer) was added and incubated at 4 °C for 1 h. The plate was washed twice with 0.1% Tween/PBS and once with PBS. The color reaction was developed by the addition of 100 mL of TMB Microwell Substrate System (BioLegend) to each well and incubation at room temperature for 5 min. The reaction was stopped by the addition of 50 µL of 20% H_2_SO_4_ to each well. The color developed was measured at optical density (OD) 450 nm using a TECAN Infinite^®^ M1000 microtiter plate reader. Background for the intensity calculation were wells coated with blocking buffer only.

### 2.6. Functional Properties

All functional experiments were performed in duplicate.

#### 2.6.1. Protein Solubility

The protein solubility was performed according to Morr, German [41] at pH 4.5 and 7.0. The soluble protein was determined using the Biuret method (550 nm), according to the AACC Approved Methods of Analysis [42], using bovine serum albumin (BSA) as standard.

#### 2.6.2. Emulsifying Capacity

The emulsifying capacity was determined according to Wang and Johnson [43] using an 1 L-reactor equipped with a stirrer and an Ultra-Turrax (IKA-Werke GmbH and Co. KG, Staufen, Germany). Mazola corn oil was added gradually (10 mL/min) to 1% (*w*/*w*) neutralized sample dispersions until a phase inversion occurred (<10 μS/cm). The volume of added oil was used to calculate the emulsifying capacity (mL oil/g sample).

$$EC=\frac{V_{oil}}{m_{sample}}$$

*EC* = emulsifying capacity (mL/g);
*V_oil_* = volume of oil used until phase inversion (mL);
*m_sample_* = weight of sample (g).

#### 2.6.3. Foaming Properties

The foaming capacity and foam stability were analyzed according to Phillips, Haque [44] using a whipping machine (Hobart N50, Hobart GmbH, Offenburg, Germany). Briefly, 5% (*w*/*v*) dispersions were adjusted to pH 7.0 and stirred for 15 min. The dispersions were whipped (580 rpm) for 8 min and the foaming capacities determined as the relation between the initial and final volume.

$$FC=\frac{V_{2}}{V_{1}}\times 100$$

*FC* = foaming capacity (%);
*V*_1_ = volume of sample solution before whipping (mL);
*V*_2_ = volume of foam after whipping (mL).

### 2.7. Sensory Analysis

#### 2.7.1. Sample Preparation

The sensory analysis was performed using the combined treated samples (HyF and FdH) and the PPI. Sample solutions (2%, *w*/*w*) were prepared with tap water and coded using three-digit random numbers.

#### 2.7.2. Sample Evaluation

The sensory evaluation was conducted according to the ISO 8587:2006 Sensory analysis—Methodology—Ranking, which compares different products according to the intensity of a given characteristic or property. First, a ten-member panel ranked attributes regarding bitterness and plant-like (pea-like/green/beany) flavor. These attributes were evaluated on a 1 (attribute not perceivable) to 7 (very strong perception) ranging scales.

### 2.8. Statistical Analysis

Complete raw data of untreated PPI, treated PPI and controls (temperature treatment) can be found in Mendeley Data files [45]. All results are expressed as mean values ± standard deviations. The microbial growth results were analyzed using the two-sample *t*-test. Further results were analyzed by one-way analysis of variance (ANOVA). The mean values were compared using Tukey’s post-hoc test. All statistical analyses, except those from the sensory analysis, were performed using OriginPro 2018b and were considered statistically significant at *p* < 0.05. A Friedman Test and Duncan Test as post-hoc test were used to analyze the results from the sensory analysis (*p* < 0.10). Ranking recording and statistical analyses of sensory data were carried out using RedJade software (RedJade Sensory Solutions, LLC, Martinez, CA, USA).

## 3. Results and Discussion

### 3.1. Microbial Growth

*L. plantarum* requires tryptophan, arginine, glutamate and branched-chain amino acids (isoleucine, leucine, valine) for growth [46]. Besides of tryptophan, PPI is a good source of all the required amino acids; thus, *L. plantarum* was able to grow both, in the PPI dispersion and hydrolyzed PPI (Table 3). However, fermentation of PPI hydrolysates resulted in significantly higher viable cell counts compared to the fermented PPI. This could be due to some release of amino acids and peptides during hydrolysis, which provide a readily available source of nutrients for *L. plantarum* growth. The hydrolysates showed slight differences in CFU after fermentation, with P_HyF showing the highest value of 9.53 Log CFU/mL followed by E_HyF and T_HyF with 9.30 Log CFU/mL and 9.17 Log CFU/mL, respectively. The pH was measured after 24 h of fermentation and was similar for all fermented samples (pH 4.5 ± 0.2). A recent study showed that lactic fermentation of hydrolyzed lupin protein isolate resulted in similar pH values regardless of the enzyme used [35].

### 3.2. Chemical Composition

The untreated PPI showed a protein content of 84.7 ± 0.1% (Table 4). The average protein content of PPI hydrolysates (83.4 ± 1.4%) was significantly higher compared to fermented PPI (79.5 ± 0.3%) and to the average of the samples produced by the combination of both treatments (76.6 ± 1.3%). The differences in protein contents might be due partial metabolism of the proteins and increase in organic acids such as lactic acid and, in lesser extent, acetic acid [46]. In addition, the ash content could be attributed to the addition of inorganic acid (hydrochloric acid) and sodium hydroxide to adjust the pH for each sample conditions.

### 3.3. Proteolysis of PPI

**Figure 1:**
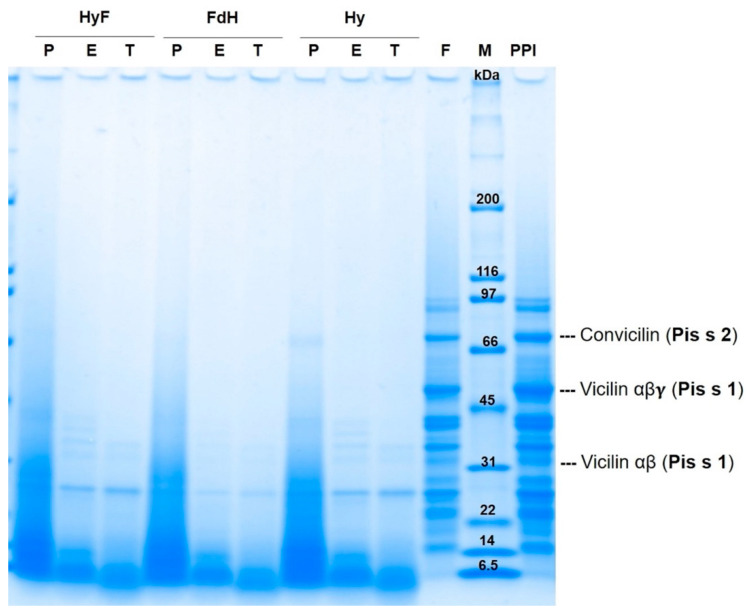
SDS-PAGE of pea protein isolate (PPI) and treated samples using *L. plantarum* and different enzymes and treatments. P: papain; E: Esperase^®^; T: trypsin; Hy: hydrolysis; F: fermented PPI; HyF: hydrolysis followed by fermentation; FdH: fermentation followed by hydrolysis; M: molecular weight standard, indicated in kilo Dalton (kDa).

The SDS-PAGE and gel filtration were performed to observe the effects of the different treatments on the pea proteins. The molecular weight distribution of the untreated PPI and treated samples is shown in Figure 1 and the positions of the main allergens are marked. The untreated PPI showed protein fractions between 97.5 and 6.5 kDa. The fermented PPI did not show major changes in the electrophoretic pattern as previously shown by García Arteaga, Leffler [33] for six lactic fermentations. The enzymatic hydrolysis facilitated significant changes in the molecular weight distribution of the respective samples with an increase in smaller peptides. This was observed in the samples that were only enzymatically hydrolyzed as well as in the samples with combined methods. The protein pattern of the sample treated with papain (P_Hy) only showed bands smaller than 40 kDa—with the exception of one band around 69.1 kDa. This band was degraded by the subsequent fermentation (P_HyF) and only bands smaller than 27 kDa were found. The proteolysis with Esperase^®^ (E_Hy) and trypsin (T_Hy) resulted in protein fractions below 40 kDa and 34 kDa, respectively. Fermentation of these hydrolysates (E_HyF and T_HyF) did not change the molecular weight distribution, while hydrolysis after fermentation (FdH) resulted in further protein degradation with protein fractions smaller than 27 kDa.

Fermentation alone did not lead to large changes in the molecular weight distribution of the respective samples, probably due to the inability of *L. plantarum* to metabolize large polypeptides [47]. Enzymatic hydrolysis enhanced the degradation of large polypeptides into smaller peptides that can be easily metabolized by *L. plantarum* [47]. Furthermore, *L. plantarum* activates peptidases with higher specificity for hydrophobic dipeptides [46]. Proteolysis is known to release hydrophobic amino acids and peptides, which then can be digested by the lactic acid bacteria.

The samples that were first fermented and then enzymatically hydrolyzed showed protein fractions below 26 kDa. One explanation might be that due to the low pH, partial acid hydrolysis occurred during fermentation, and the enzymes then further broke down these hydrolyzed fractions.

#### 3.3.1. Effect of Combined Methods on Pea Protein Allergens

A protein band at 63–80 kDa [4,18,48,49] could represent the Pis s2 allergen. In the present study, a protein band around 70.9 ± 0.9 kDa was found in the untreated PPI and with reduced intensity in the fermented PPI; this fraction could correspond to the Pis s2. The reduction could be due to a reduction in protein solubility (as explained later in Section 3.5.1) rather than to a proteolytic effect of fermentation with *L. plantarum*. Furthermore, P_Hy also showed this allergen fraction with a slightly lower intensity than the untreated PPI. This could explain that papain alone was not able to cleave this fraction.

Protein bands found around 50.1 ± 0.8 kDa and 31.91 ± 0.5 kDa could correspond to the Pis s1 of the mature vicilin (αβγ) and its proteolytic fraction (αβ), respectively. The Pis s1 αβγ was present in the untreated PPI and with less intensity in the fermented PPI. Its proteolytic fraction was present in the untreated PPI, fermented PPI, E_Hy, T_Hy, E_HyF, and T_HyF.

#### 3.3.2. Effect of Combined Methods on the Degree of Hydrolysis

Both trypsin and Esperase^®^ are serine endoproteases, with trypsin having specificity for basic residues, such as lysine and arginine derivatives [50] and Esperase^®^ having a broader specificity, such as for both hydrophobic and hydrophilic residues [51]. The latter might explain the higher DH of all Esperase^®^ treated samples (Table 5). Papain cleaves peptide bonds C-terminal of glycine and cysteine residues among others [52]. Glycine and cysteine residues might interfere with the OPA agent giving unstable and weak signals [40,53]. This effect might have been the reason why papain treated samples showed lower DH compared to other hydrolyzed samples even when the electrophoretic results showed significant changes.

Furthermore, the combination of fermentation after enzymatic hydrolysis significantly increased the DH value compared to the untreated PPI, the fermented PPI and the enzymatic treated sample. This could be related to the aforementioned ability of *L. plantarum* to take up the smaller peptides released after enzymatic hydrolysis. However, P_FdH and E_FdH did not show significant differences to P_Hy and E_Hy samples, respectively. In the case of P_FdH, this could be due to the higher exposure of cysteine residues interfering with the measurement; in the case of E_FdH, this could be due to protein agglomeration promoted by fermentation, which hides the cleavage site for Esperase^®^.

### 3.4. Reaction of Polyclonal Antibodies with PPI

**Figure 2:**
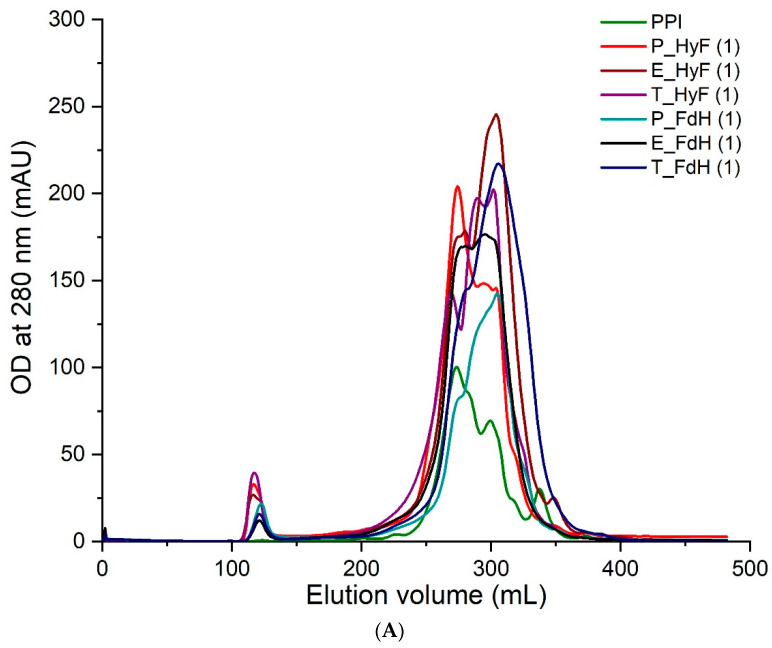

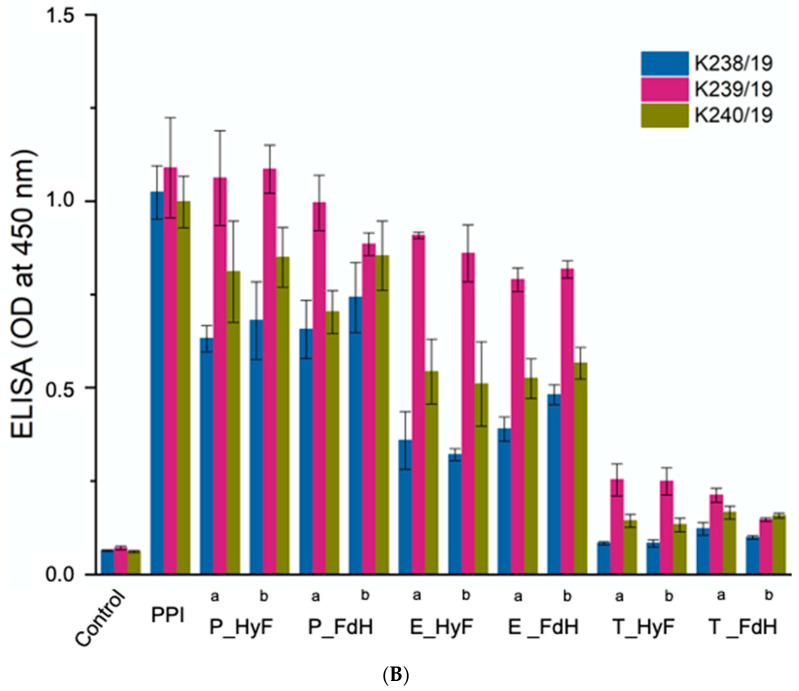
Protein size distribution and ELISA of pea protein isolate (PPI) and its hydrolysates by means of (**A**) gel filtration and (**B**) ELISA of total protein using three immunized rabbit sera. The sample replicates were analyzed independently (a and b). P: papain; E: Esperase^®^; T: trypsin; Hy: hydrolysis; HyF: hydrolysis followed by fermentation; FdH: fermentation followed by hydrolysis.

**Figure 3:**
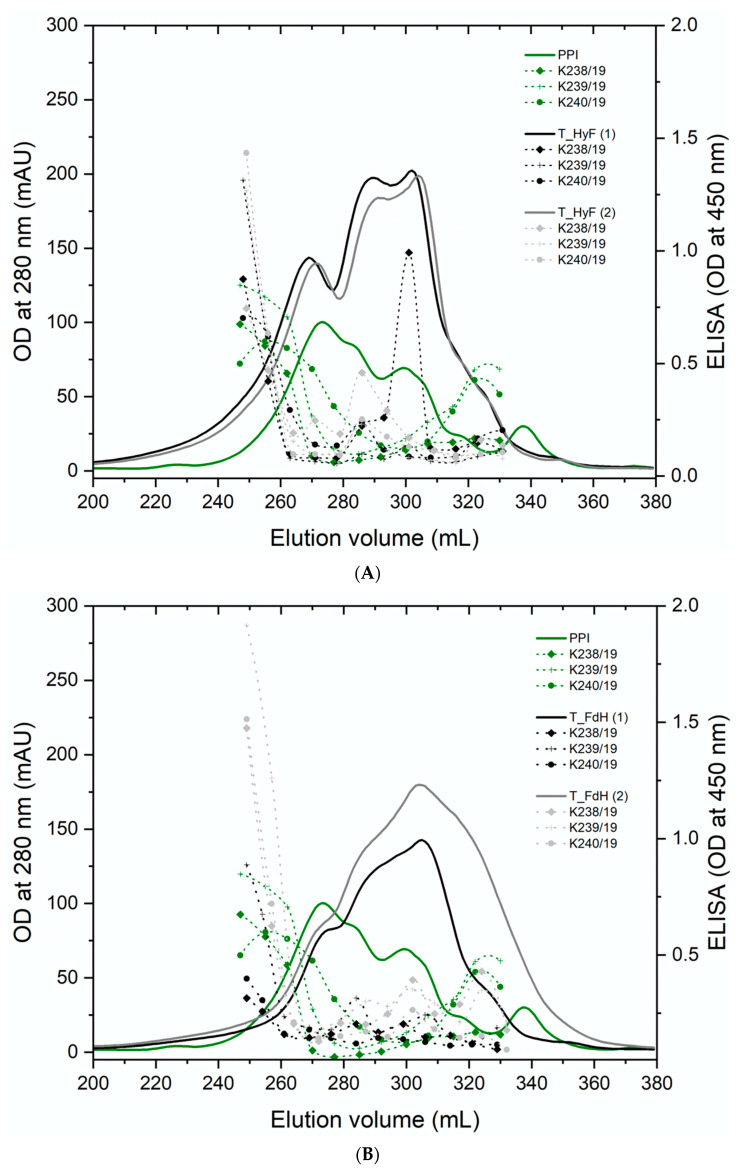
Gel filtration and ELISA results from trypsin treated protein isolates by combination methods of (**A**) enzymatic hydrolysis followed by fermentation (T_HyF) and (**B**) fermentation followed by enzymatic hydrolysis (T_FdH).

The soluble proteins from all samples were separated by gel filtration and individual fractions analyzed by ELISA using three individual polyclonal rabbit sera raised against PPI. The results from all treated samples showed a compelling degradation towards lower molecular weight proteins (Figure 2A). ELISA analyses of the total protein (Figure 2B) and individual fractions gave a reduced immunogenicity for all samples. In particular, trypsin-treated samples showed a reduced antibody reactivity to background levels (Figure 3A,B). Since the three polyclonal sera used for the ELISA showed different binding profiles for the individual proteins, it can be concluded that the soluble proteins are no longer recognized by the antibodies. The only exception are the eluted fractions containing higher molecular weight proteins, which are certainly resistant to the treatment applied. The ELISA results for the total protein showed that also the overall signal is significantly reduced in those preparations with the highest degradation. Therefore, the fraction of high molecular weight immunogenic proteins may be lower than suggested by the ELISA values of the high molecular weight fractions.

These SDS-PAGE results in combination with the results from the gel filtration and ELISA show that the combination of enzymatic hydrolysis and fermentation degrades pea proteins to a higher degree. Reduced reactivity with the antibody sera could imply reduction of the allergic potential of pea protein preparations. Fermentation followed by enzymatic hydrolysis was particularly successful, as it seemed to degrade all major potential pea allergens. However, the reduction in allergenicity needs to be confirmed by further immunological studies, such as prick tests.

### 3.5. Functional Properties

Changes in the molecular weight distribution of proteins cause changes in the exposed hydrophobic and ionizable groups as well as in the ability of the proteins to aggregate, which can influence the functional properties [54]. Therefore, the effect of protein degradation on functional properties were studied in detail.

#### 3.5.1. Protein Solubility

The results of the protein solubility analyses are shown in Table 6; these results correlate strongly with the DH values. At acidic pH (pH 4.5), the untreated and fermented PPI were significantly different from all other samples. Samples treated with Esperase^®^ showed the highest protein solubility of up to 66%, whereas the protein solubility of papain and trypsin treated samples was also significantly increased. The fermentation followed by enzymatic hydrolysis was most effective in increasing solubility at acidic pH.

At neutral pH, the fermented PPI showed significant lower protein solubility compared to the untreated PPI. The PPI showed similar protein solubility to P_Hy and P_HyF, whereas the P_FdH was significantly different. The papain and trypsin treated samples showed the highest protein solubility when the fermentation step was followed by enzymatic hydrolysis. Among the samples that were only hydrolyzed or were hydrolyzed and then fermented, a significant difference in protein solubility could not be measured. However, the samples treated with Esperase^®^ were significantly different from each other and from the untreated PPI. The lower solubility of E_FdH compared to E_HyF could be explained by an increase in insoluble aggregates due to acid denaturation during fermentation, which hinders the Esperase^®^ activity to cleave on specific protein sites.

Other studies have shown negative or no effect of fermentation on the protein solubility. This has been attributed to changes in the protein surface, surface charge and the LAB cell surface, which might promote hydrophobic interactions [33,47,55]. Thus, the improvement in the protein solubility of treated samples is certainly due to enzymatic hydrolysis.

#### 3.5.2. Emulsifying Capacity

Results from emulsifying capacity are shown in Table 6. The untreated PPI showed the highest emulsifying capacity with 725 mL/g, followed by T_FdH with 700 mL/g. In contrast, the fermented PPI and the E_HyF showed the lowest emulsifying capacity with 310 mL/g and 300 mL/g, respectively. The difference among the results of the treated samples could be due to different changes in protein conformation, peptide release, and their interactions with other components such as microbial cells, which could reduce the amphiphilic character of the proteins [56]. Moreover, the ratio albumin/legumin/vicilin, the presence of polar lipids and partial denaturation have also been shown to affect emulsifying capacity [57,58,59,60]. Although all treated samples had lower emulsifying capacities than the untreated PPI, the emulsifying capacity of the treated samples is still in a good range to be used as food ingredient. A high DH is known to impair emulsifying capacities [54], and although there was no correlation between the DH value and the emulsifying capacity, the sample with the highest DH (E_HyF) showed the lowest emulsifying capacity.

#### 3.5.3. Foaming Capacity

A foam is a dispersion of air in water. The effect of proteins in foam formation is similar to the one in forming emulsions. Their amphiphilic character allows proteins to interact with the hydrophobic and hydrophilic fractions of air and water, respectively, during whipping, reducing surface tension. Similarly, the foaming capacity depends on different factors such as protein fractions ratio, pH of the solutions, and lipid content [61,62].

The foaming capacities of untreated and fermented PPI were not significantly different with 840% and 807%, respectively. On the other hand, all other treated samples showed a significantly improved foaming capacity, with the highest foaming capacity found in the fermented and subsequently hydrolyzed samples. Of the treated samples, the trypsin samples showed the highest capacities.

### 3.6. Sensory Analysis

Although products containing pea proteins are increasing, the characteristic pea off-flavors remain a major challenge. In addition to naturally occurring off-flavors, PPI treatment can lead to changes in the flavor and taste profile. It is known that enzymatic hydrolysis can increase the bitterness of protein preparations from legumes, whereas fermentation of legumes promotes the degradation and formation of aroma compounds.

**Figure 4:**
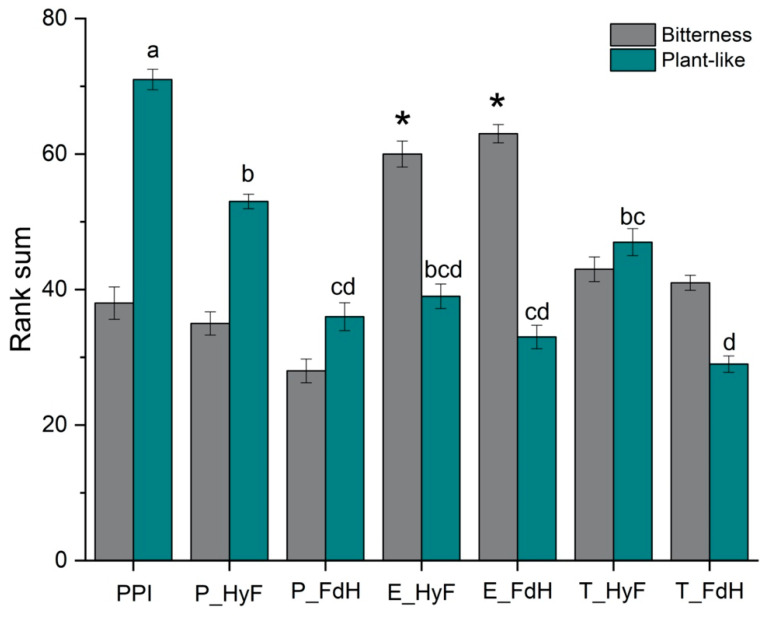
Results are expressed as sum of ranks ± standard deviation (n = 11). Rank sums marked with different letters indicate significant differences (Duncan’s, *p* < 0.1). An asterisk (*) indicate significant differences to the untreated PPI (Duncan’s, *p* < 0.1). PPI: pea protein isolate; P: papain; E: Esperase^®^; T: trypsin; HyF: hydrolyzed followed by fermentation; FdH: fermentation followed by hydrolysis.

The bitterness of the untreated PPI compared to those treated with papain or trypsin was not significantly different (Figure 4). However, the bitterness ranking was the highest after treatment with Esperase^®^ and was significantly higher than that of the untreated PPI. Although fermentation enhanced further hydrolysis (Section 3.3), the peptidases from *L. plantarum* may not be sufficient to completely cleave hydrophobic residues.

As expected, the untreated PPI was ranked highest for plant-like off-flavor, while this attribute was significantly reduced for all combined samples. The samples fermented prior to enzymatic hydrolysis showed the strongest reduction of the plant-like off-flavor, where T_FdH received the lowest rank.

## 4. Conclusions

Various studies have investigated the effects of enzymatic hydrolysis and fermentation on pea proteins; however, to the best of our knowledge, a combination of both methods has not yet been investigated. Our study shows that the order of combination of both methods can have a significant impact on the proteins, their immunological and functional properties, as well as the characteristic off-flavors of PPI. The fermentation of PPI followed by enzymatic hydrolysis showed stronger protein degradation and an effect on functionality of the proteins as well as a reduction of off-flavors. The SDS-PAGE and gel filtration showed a significant reduction in the proteins molecular weight by enzymatic digestion. Analyses of the individual size fractions showed a reduced immunogenicity using three different polyclonal sera in ELISA. However, further in vivo tests are required to confirm that treated PPI will be tolerated better by allergic or sensitized individuals at those amounts corresponding to the daily consumption in protein-enriched food. The increase in protein solubility, especially in acidic conditions, suggests that treated pea proteins can be used to increase the protein content in different food products. The reduction of pea off-flavors could allow the increase of protein content without hindering the acceptance by consumers; the application in different products and their acceptance still need to be investigated. The combination of treatments can be a promising method to be used in the food industry to enhance pea protein isolate functionality and neutralize off-flavors, and could significantly lower the allergenicity.

## Figures and Tables

**Table 1:** Optimal conditions of commercial enzyme preparations and microorganism.

Enzyme/Microorganism	Amount	Temp. (°C)	pH Value (-)	Activity	Origin
Papain	0.1% E/S	65	7	Cysteine Endoprotease	Papaya latex
Esperase^®^ 8.0 L	0.5% E/S	65	8	Serine Endoprotease	*Bacillus* sp.
Trypsin	0.1% E/S	50	8	Serine Endoprotease	Bovine pancreas
*Lactobacillus plantarum*	7 Log CFU/mL	30	6.5	Anaerobe	Pickled cabbage

E/S: enzyme/substrate ratio; Temp: temperature.

**Table 2:** Treatment sample code.

Sample Code	Treatment 1	Treatment 2
Untreated PPI	-	-
Fermented PPI	*L. plantarum*	-
P_Hy	Papain	-
P_HyF	Papain	*L. plantarum*
P_FdH	*L. plantarum*	Papain
E_Hy	Esperase^®^	-
E_HyF	Esperase^®^	*L. plantarum*
E_FdH	*L. plantarum*	Esperase^®^
T_Hy	Trypsin	-
T_HyF	Trypsin	*L. plantarum*
T_FdH	*L. plantarum*	Trypsin

**Table 3:** Colony forming units (CFU) after inoculation and 24 h of fermentation.

	Log CFU/mL	
	0 h	24 h
Fermented PPI	7.40 ± 0.10 ^a^	8.89 ± 0.09 ^b^*
P_HyF	7.41 ± 0.03 ^a^	9.53 ± 0.45 ^b^
E_HyF	7.37 ± 0.15 ^a^	9.30 ± 0.01 ^b^
T_HyF	7.39 ± 0.01 ^a^	9.17 ± 0.03 ^b^

Results are expressed as means ± standard deviation (n = 2). Means marked with different letters indicate significant differences between 0 h and 24 h within same row (two-sample *t*-test, *p* < 0.05). Means marked with an asterisk (*) indicate significant differences between fermented pea protein isolate (PPI) and fermented hydrolysates (HyF) within the same column (One-way ANOVA, Tukey, *p* < 0.05). P: papain; E: Esperase^®^; T: trypsin.

**Table 4:** Chemical composition of untreated and treated pea protein isolates.

Samples	Dry Matter (%)	Protein Content (%) *	Ash Content (%) *
Untreated PPI	96.6 ± 0.3 ^a^	84.7 ± 0.1 ^a^	5.2 ± 0.5 ^a^
Fermented PPI	94.9 ± 0.6 ^b^	79.5 ± 0.3 ^b^	6.9 ± 0.1 ^bde^
P_Hy	92.7 ± 0.7 ^c^	84.9 ± 0.1 ^a^	5.5 ± 0.1 ^ac^
P_HyF	97.6 ± 0.1 ^a^	78.3 ± 0.2 ^c^	7.2 ± 0.5 ^b^
P_FdH	96.7 ± 1.2 ^a^	78.0 ± 0.2 ^c^	5.9 ± 0.8 ^cd^
E_Hy	94.8 ± 1.0 ^b^	82.0 ± 0.8 ^c^	6.3 ± 0.2 ^b^
E_HyF	97.6 ± 0.2 ^a^	74.9 ± 0.1 ^d^	9.3 ± 0.2 ^c^
E_FdH	96.6 ± 0.3 ^a^	76.4 ± 0.3 ^e^	8.4 ± 0.5 ^c^
T_Hy	92.5 ± 0.9 ^c^	83.3 ± 0.1 ^c^	5.9 ± 0.5 ^ab^
T_HyF	97.9 ± 0.5 ^a^	75.8 ± 0.3 ^d^	7.9 ± 1.1 ^e^
T_FdH	96.1 ± 1.9 ^a^	76.1 ± 0.2 ^d^	7.8 ± 0.8 ^e^

Results are expressed as means ± standard deviation (n = 4). Means marked with different letters within one column indicate significant differences between treated samples from each enzyme and the untreated pea protein isolate (PPI) and fermented PPI (Tukey, *p* < 0.05). P: papain; E: Esperase^®^; T: trypsin; Hy: hydrolysis; HyF: hydrolysis followed by fermentation; FdH: fermentation followed by hydrolysis. * based on the dry matter content.

**Table 5:** Degree of hydrolysis (%) of untreated and treated pea protein isolates.

Samples	DH [%]
Untreated PPI	1.88 ± 0.14 ^a^
Fermented PPI	1.32 ± 0.05 ^b^
P_Hy	3.73 ± 0.08 ^c^
P_HyF	5.48 ± 0.16 ^d^
P_FdH	3.92 ± 0.44 ^c^
E_Hy	9.57 ± 0.46 ^c^
E_HyF	10.76 ± 0.15 ^d^
E_FdH	9.98 ± 0.37 ^c^
T_Hy	6.86 ± 0.06 ^c^
T_HyF	9.22 ± 0.20 ^d^
T_FdH	9.26 ± 0.27 ^d^

Results are expressed as means ± standard deviation (n = 4). Means marked with different letters within one column indicate significant differences treated samples from one enzyme and the untreated pea protein isolate (PPI) and the fermented PPI (Tukey, *p* < 0.05). P: papain; E: Esperase^®^; T: trypsin; Hy: hydrolysis; HyF: hydrolysis followed by fermentation; FdH: fermentation followed by hydrolysis.

**Table 6:** Functional properties of untreated and treated pea protein isolates.

Samples	Protein Solubility [%]		Emulsifying Capacity	Foaming Capacity
	pH 4.5	pH 7.0	(mL/g)	(%)
Untreated PPI	6.98 ± 0.47 ^a^	40.26 ± 0.81 ^a^	725 ± 8 ^a^	840 ± 8 ^a^
Fermented PPI	5.72 ± 0.44 ^a^	10.72 ± 1.67 ^b^	310 ± 13 ^b^	807 ± 3 ^a^
P_Hy	31.19 ± 1.24 ^b^	43.64 ± 1.99 ^ac^	465 ± 18 ^c^	1234 ± 56 ^b^
P_HyF	35.87 ± 1.12 ^c^	42.85 ± 1.38 ^ac^	398 ± 21 ^d^	1190 ± 17 ^b^
P_FdH	38.12 ± 1.69 ^c^	47.37 ± 4.42 ^c^	383 ± 10 ^d^	1335 ± 73 ^c^
E_Hy	60.01 ± 1.25 ^b^	61.52 ± 1.01 ^c^	391 ± 10 ^c^	1261 ± 67 ^b^
E_HyF	63.74 ± 1.46 ^c^	74.95 ± 2.65 ^d^	300 ± 14 ^b^	985 ± 33 ^c^
E_FdH	66.55 ± 1.64 ^d^	67.28 ± 2.76 ^e^	450 ± 4 ^d^	1576 ± 22 ^d^
T_Hy	42.95 ± 7.04 ^b^	50.94 ± 2.19 ^c^	670 ± 31 ^c^	1993 ± 53 ^b^
T_HyF	48.89 ± 1.87 ^bc^	52.55 ± 1.20 ^c^	664 ± 24 ^c^	1934 ± 150 ^b^
T_FdH	51.31 ± 0.44 ^c^	63.08 ± 2.22 ^d^	705 ± 12 ^ac^	2575 ± 47 ^c^

Results are expressed as means ± standard deviation (n = 4). Means marked with different letters within one column indicate significant differences between treated samples from one enzyme and the untreated pea protein isolate (PPI) and the fermented PPI (Tukey, *p* < 0.05). P: papain; E: Esperase^®^; T: trypsin; Hy: hydrolysis; HyF: hydrolysis followed by fermentation; FdH: fermentation followed by hydrolysis.
